# Supplementary material for: AffyMAPSDetector: a software tool to characterize Affymetrix GeneChip™ expression arrays with respect to SNPs
Source: BMC Bioinformatics. 2007 Jul 30;8:276. doi: 10.1186/1471-2105-8-276 (PMC1959249; doi:10.1186/1471-2105-8-276)
Supplement: Additional file 13 — AffyMAPSDetector v1 source code. AffyMAPSDetector source code. [file 1471-2105-8-276-S13.zip › AffyMAPSDetector_v1_src/doc/index-files/index-20.html]

W-Index


|  |  |  |  |
| --- | --- | --- | --- |
| **Overview** \| Package \| Class \| Use \| **Tree** \| **Deprecated** \| **Index** \| **Help** | | |  |
| **PREV LETTER**   **NEXT LETTER** | **FRAMES**    **NO FRAMES**     **All Classes** |  |  |


A B C D E F G H I J L M N O P R S T V W X Y 

---


## **W**

**WEB\_GENE\_INFO\_FILENAME** - Static variable in class affychip.snpdetector.Constant: **WEB\_SNP\_INFO\_FILEMANE** - Static variable in class affychip.snpdetector.Constant: **WIZARD\_WINDOW\_SIZE** - Static variable in class jwf.Wizard: **Wizard** - class jwf.Wizard.: This class controls a wizard. **Wizard(Map)** - Constructor for class jwf.Wizard: **Wizard()** - Constructor for class jwf.Wizard: Creates a new wizard. **WizardAdapter** - class jwf.WizardAdapter.: This class provides means of abreviating work when using the WizardListener allowing the developer to implement only the needed methods **WizardAdapter()** - Constructor for class jwf.WizardAdapter: Creates a new instance of WizardAdapter **WizardContext** - class jwf.WizardContext.: An instance of this class is maintained by the Wizard. **WizardContext()** - Constructor for class jwf.WizardContext: **WizardListener** - interface jwf.WizardListener.: Implement this interface to be able to receive wizard related events. **WizardPanel** - class jwf.WizardPanel.: The base class used for implementing a panel that is displayed in a Wizard. **WizardPanel()** - Constructor for class jwf.WizardPanel: A default constructor. **WizardUtils** - class jwf.WizardUtils.: Description: This is a general purpose utility class that is a convenient place for wizard-wide utitilites. **WizardUtils()** - Constructor for class jwf.WizardUtils: **wizardCancelled(Wizard)** - Method in class affychip.snpdetector.ui.FileLoaderWizardDialog: **wizardCancelled(Wizard)** - Method in class affychip.snpdetector.util.GeneAnnotLoaderWizard: **wizardCancelled(Wizard)** - Method in class jwf.WizardAdapter: Called when the wizard is cancelled. **wizardCancelled(Wizard)** - Method in interface jwf.WizardListener: Called when the wizard is cancelled. **wizardContext** - Variable in class jwf.WizardPanel: The context of the wizard process. **wizardFinished(Wizard)** - Method in class affychip.snpdetector.ui.FileLoaderWizardDialog: **wizardFinished(Wizard)** - Method in class affychip.snpdetector.util.GeneAnnotLoaderWizard: **wizardFinished(Wizard)** - Method in class jwf.WizardAdapter: Called when the wizard finishes. **wizardFinished(Wizard)** - Method in interface jwf.WizardListener: Called when the wizard finishes. **wizardPanelChanged(Wizard)** - Method in class affychip.snpdetector.ui.FileLoaderWizardDialog: Called when a new panel has been displayed in the wizard. **wizardPanelChanged(Wizard)** - Method in class affychip.snpdetector.util.GeneAnnotLoaderWizard: **wizardPanelChanged(Wizard)** - Method in class jwf.WizardAdapter: Called when a new panel has been displayed in the wizard. **wizardPanelChanged(Wizard)** - Method in interface jwf.WizardListener: Called when a new panel has been displayed in the wizard.

---


|  |  |  |  |
| --- | --- | --- | --- |
| **Overview** \| Package \| Class \| Use \| **Tree** \| **Deprecated** \| **Index** \| **Help** | | |  |
| **PREV LETTER**   **NEXT LETTER** | **FRAMES**    **NO FRAMES**     **All Classes** |  |  |


A B C D E F G H I J L M N O P R S T V W X Y 

---
